# Role of Herbal Plants for Treatment of Coronary Artery Disease; A Review: Herbalism for Coronary Artery Disease

**DOI:** 10.31661/gmj.v14i.3737

**Published:** 2025-02-21

**Authors:** Leila Azizkhani, Sharareh Jahangiri, Seyedeh Mahya Dehestani, Hamid Hojati, Yahya Ebrahimi

**Affiliations:** ^1^ Clinical Care Research Center, Research Institute for Health Development, Kurdistan University of Medical Sciences, Sanandaj, Iran; ^2^ Department of Anesthesiology, Tehran University of Medical Science, Tehran, Iran; ^3^ Manchester University, United Kingdom; ^4^ Department of Nursing, Golestan University of Medical Sciences, Gorgan, Iran; ^5^ Department of Cardiology, School of Medicine, Shahid Madani Hospital, Lorestan University of Medical Sciences, Khorramabad, Iran

**Keywords:** Cardiovascular Diseases, Medicinal Plants, Coronary Artery Disease, Sedatives, Anti-Anxiety Agents

## Abstract

**Background:**

Chronic stress is recognized as a significant risk factor for cardiovascular disease and can worsen the condition of patients with coronary artery disease (CAD). Traditional medicine is used to reduce stress and manage various diseases, with a particular focus on cardiovascular health. This study aimed to investigate the role of herbal supplements in the management of coronary artery disease (CAD), focusing on their potential to alleviate symptoms, enhance heart health, and reduce the risk of cardiovascular complications in CAD patients, alongside the mechanisms of action underlying their effects.

**Materials and Methods:**

This comprehensive review evaluates the impact of medicinal plants on stress and cardiovascular disease in research and studies with coronary artery disease. Scientific articles were sourced from databases including PubMed, Google Scholar, SID, and ScienceDirect, focusing on publications from 2000 to 2024. The selection criteria encompassed research and studies involving various dosages of medicinal plants under different conditions. Only articles in Persian and English were included, excluding those in other languages. Data from the selected articles were extracted, specifically focusing on medicinal plants effective as cardiac tonics within different regions of Iran.

**Results:**

The findings indicate that various medicinal plants, including marjoram (Origanum majorana), borage (Borago officinalis), lavender (Lavandula angustifolia), valerian (Valeriana officinalis), hops (Humulus lupulus), hawthorn (Crataegus spp.), rosemary (Rosmarinus officinalis), chamomile (Matricaria chamomilla), dracocephalum (Dracocephalum moldavica), mint (Mentha spp.), green tea (Camellia sinensis), and damask rose (Rosa damascena), have been employed for stress relief and the management of CAD, particularly in the context of mitigating cardiovascular disease risk factors.

**Conclusion:**

The combination of medicinal plants with other treatments can be an effective strategy to reduce stress and enhance cardiovascular health in coronary artery disease patients. Due to their low risk and broad therapeutic benefits, these plants can improve patients’ quality of life when used alongside conventional therapies.

## Introduction

Cardiovascular disease (CVD) is one of the primary causes of death globally and a significant factor in overall mortality to the burden of disease in various populations [1]. These diseases are influenced by a variety of factors, including hypertension, hypercholesterolemia, diabetes, unhealthy lifestyle, stress, and tobacco use. In addition, factors such as family history, age and gender can also increase the risk of developing cardiovascular disease, underscoring the critical need for effective prevention and management strategies [2].

Coronary artery disease (CAD) is a major global cause of mortality [3]. It occurs when the coronary arteries become blocked or narrowed, resulting in reduced blood flow to the heart muscle, which can adversely affect heart function [4]. CAD is primarily caused by atherosclerosis, in which excess fat and cholesterol build up in the artery walls, causing them to narrow [4]. This condition, known as myocardial ischemia, impairs the supply of oxygen and nutrients to the heart, causing chest pain and potentially leading to a heart attack [5].

CAD can be divided into two main types: "stable ischemic heart disease" and "acute coronary syndrome," each with distinct symptoms that require diagnosis by a cardiologist [6]. The most critical complication of CAD is myocardial infarction, which requires immediate treatment to prevent myocardial death and ensure patient survival. Other complications include arrhythmias, cardiac shock, heart failure, and cardiac arrest [4][5][6]. CAD is associated with several risk factors such as stress, obesity, hypertension, diabetes, smoking, and family history. In addition, an unhealthy diet high in saturated and trans fats and salt contributes to the disease. Preventive and therapeutic measures include lifestyle changes such as weight loss, smoking cessation, increased physical activity, stress management, and dietary improvements [7].

Stress is the body's physiological and psychological response to changes and pressures in life. While these responses can be beneficial in threatening situations, their frequent or severe occurrence can adversely affect an individual's physical and mental health and contribute to the onset of cardiovascular disease, particularly coronary heart disease [8]. Cardiovascular diseases have been recognized since ancient times, with evidence found in Egyptian mummies.

Key milestones, such as William Harvey’s discovery of blood circulation in the 17th century and the invention of the stethoscope in the 19th century, contributed significantly to the understanding and diagnosis of these conditions. In the 20th and 21st centuries, breakthroughs in genetics, molecular biology, and modern therapeutic methods have greatly advanced strategies for the prevention and treatment of cardiovascular diseases [9]. Stress resulting from various factors, including a history of CVD and social relationships, can lead to physical complications. These problems result from the body's physiological responses, such as increased heart rate and the release of hormones such as adrenaline [9]. Treatment of coronary artery disease includes pharmacological therapies and invasive procedures. Medications such as statins, antihypertensives, and anticoagulants are used to manage the condition. In cases requiring invasive intervention, the treatment of coronary artery disease involves balloon angioplasty to open blocked arteries, stent placement to prevent reclosure of the arteries, and coronary artery bypass surgery to bypass blocked areas. In some cases, heart valve repair or replacement surgery may also be performed [10][11]. Strategies such as cognitive behavioral therapy (CBT), mindfulness, and relaxation techniques (such as yoga) can also be used as part of a comprehensive approach to stress management and prevention of coronary artery disease [12].

Pharmacological and invasive treatments for coronary artery disease may cause complications like myopathy, bleeding, and hypotension, requiring close monitoring [12]. The use of medicinal plants as a complementary approach to disease management has received considerable attention in recent years [13][14][15][16]. Numerous medicinal plants possess anti-inflammatory, antioxidant, antihypertensive, and anti-stress properties that may contribute to improve heart health and reduced risk of heart disease [17][18][19]. While medicinal plants can serve as a valuable adjunct in the management of cardiovascular disease such as saffron, borage, chamomile, valerian, and ginger which their use should be supervised by a physician and integrated with conventional medical treatments. These plants can be an essential component of a holistic strategy aimed at maintaining heart health, offering several advantages: first, they provide a complementary treatment alongside conventional therapies; second, they can reduce the side effects commonly associated with synthetic medications; third, the diversity of these plants offers a promising avenue for discovering new and more effective treatments; and fourth, they serve as a preventive measure for reducing the risk of heart diseases, especially in individuals at higher risk [15][16][17][18][19].

In traditional Iranian medicine, medicinal plants such as marjoram, borage, lavender, valerian, hops, hawthorn, rosemary, chamomile, mint, green tea, and damask rose are believed to possess sedative, anti-anxiety, anti-inflammatory, blood pressure-lowering, and heart function-enhancing properties. These attributes are thought to contribute to stress reduction and cardiovascular health improvement, thereby potentially decreasing the risk of heart diseases [12][13][14][15][16][17][18][19].

The aim of this study is to investigate the role of herbal supplements in the management of coronary artery disease (CAD), focusing on their potential to alleviate symptoms, improve heart health, and reduce the risk of cardiovascular complications in CAD patients, along with the underlying mechanisms of their effects.

## Materials and Methods

**Table:** Table1. The Strategies of Medicinal Plants on Cardiovascular Diseases in Iran

**Database**	**Search Strategy **	**Initial Articles **	**Selected Articles **
**PubMed**	"medicinal plants" AND "cardiovascular disease"	9	8
**Google Scholar**	"stress AND medicinal plants" AND "coronary artery disease"	10	9
**SID**	"herbal medicine" AND "heart disease"	8	6
**ScienceDirect**	"plant extracts AND heart tonic"	5	4
**The final conclusion for the strategies of search**	In this review, a total of 27 records were retrieved from databases. After a thorough evaluation, 12 studies were found to meet the inclusion criteria. Additionally, 12 reports related to these studies were analyzed and included in the review. It is worth noting that all included studies were in English.		

**Figure-1:**
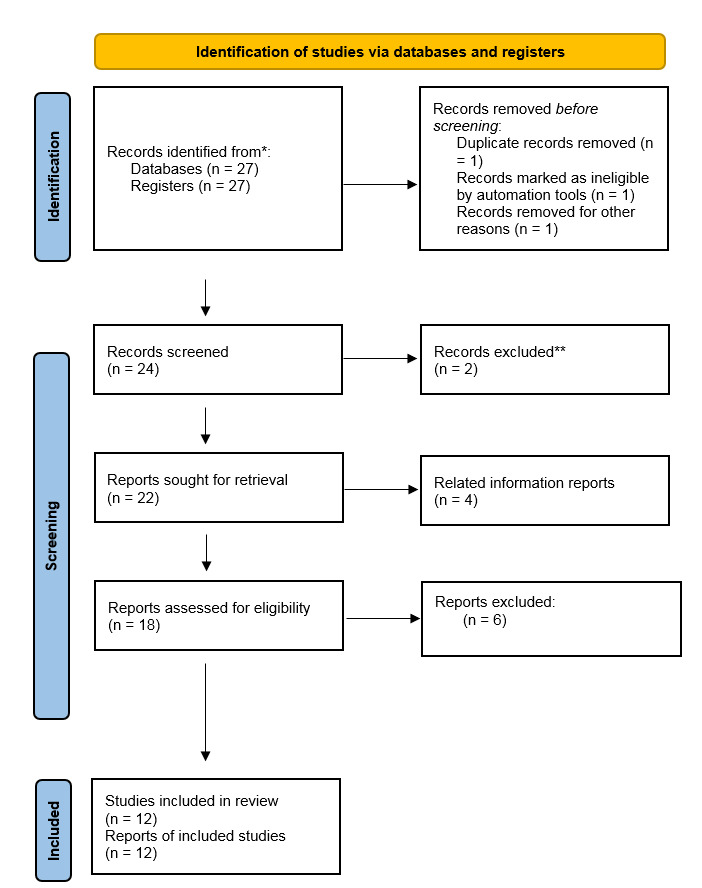
Figure 1. Search strategy flowchart

A comprehensive review the study was carried out to assess the impact of medicinal plants on stress and cardiovascular disease in patients with coronary artery disease. This review included a variety of scientific sources and reputable articles retrieved from databases such as PubMed, Google Scholar, SID, and ScienceDirect. Study selection criteria included clinical and experimental research, as well as different doses of medicinal plants under different conditions. The search period was from 2000 to 2024. Only articles in Persian and English were selected for review, and articles in other languages were excluded.

### Data Analysis

After selecting the final articles, data related to medicinal plants effective on cardiac tonic were extracted from the ethnobotany of different parts of Iran. The obtained information was classified in the Table-1 and Figure-1.

In the Figure below (Figure-1), the steps of selecting and reviewing articles are shown in Figure-1. In this review, 27 records were identified from databases and 27 records from registers. After a thorough assessment, 12 studies were deemed eligible for inclusion in the review. Additionally, 12 reports of the included studies were evaluated and utilized. The results are presented in Table-1.

The strategies of medicinal plants on cardiovascular diseases in Iran are specified in Table-1. Analysis of Indexing and Publishers of the Reviewed Journals are specified in below.

### Review of Journal Indexing Status

7 journals are indexed in reputable databases, including PubMed, Google Scholar, and ScienceDirect.

2 journals (Modern Care Journal and Journal of Contemporary Medical Sciences) are only indexed in SID.

1 journal (Biomedical Research and Therapy) is indexed in SID and published by BIOMEDPRESS, Ho Chi Minh, Vietnam.

### Analysis of Journal Publishers

Elsevier publishes 2 journals (Journal of Ethnopharmacology and Clinical Nutrition ESPEN).

Springer publishes 2 journals (Inflammopharmacology and Naunyn-Schmiedeberg's Archives of Pharmacology).

Hindawi Publishing Corporation publishes 1 journal (Evidence-Based Complementary and Alternative Medicine).

Dove Medical Press (a subsidiary of Taylor & Francis Group) publishes 1 journal (Journal of Pain Research).

De Gruyter publishes 1 journal (Journal of Complementary and Integrative Medicine).

BIOMEDPRESS, Ho Chi Minh, Vietnam publishes 1 journal (Biomedical Research and Therapy).

2 journals do not have a specified publisher (Modern Care Journal and Journal of Contemporary Medical Sciences).

### Analysis of Journal Credibility and Accessibility

7 journals are published by internationally recognized publishers and indexed in major academic databases, indicating high scientific credibility.

3 journals, which are only indexed in SID, likely have a more limited scientific impact.

### Ethics Approval

This study was performed in line with the principles of the Declaration of Helsinki.

## Results

**Table:** Table2. Medicinal Plants Effective in Reducing Stress and Managing Coronary Artery Disease according to Iranian Traditional Medicine

**Persian Name**	**Common Name**	**Scientific Name**	**Family**	**Mechanism**	**Ref**
Marzanjoosh	Marjoram	*Origanum majorana*	Lamiaceae	Sedative, anti-anxiety, anti-inflammatory and antispasmodic effects. Helps to reduce stress and blood pressure.	[20]
Gavzaban	Borage	*Borago officinalis*	Boraginaceae	Anti-inflammatory, antioxidant, anxiety reduction and heart function improvement properties.	[21]
Ostokhodoos	Lavender	*Lavandula angustifolia*	Lamiaceae	Sedative, anti-anxiety, blood pressure-lowering, and stress-relieving effects	[22]
Sonbolatib	Valerian	*Valeriana officinalis*	Valerianaceae	Sedative, anti-anxiety, sleep improvement and stress reduction properties	[23]
Razak	Hops	*Humulus lupulus*	Cannabaceae	Sedative, anti-anxiety and anti-inflammatory effects, helps improve sleep and reduce stress	[24]
Zalzalak	Hawthorn	*Crataegus monogyna*	Rosaceae	Anti-inflammatory, antioxidant, cardiovascular, and blood pressure-lowering effects	[25]
Rosemary	Rosemary	*Rosmarinus officinalis*	Lamiaceae	Antioxidant, anti-inflammatory, blood circulation and heart function improvement effects	[26]
Babboneh	Chamomile	*Matricaria chamomilla*	Asteraceae	Sedative, anti-inflammatory, anti-anxiety, and antihypertensive effects	[27]
Badranjboyeh	Dracocephalum	Echium amoenum	Lamiaceae	Decreases stress, blood pressure, and vascular inflammation	[28]
Naana	Mint	*Mentha spicata*	Lamiaceae	It reduces stress, enhances circulation, and lowers blood pressure, helping to decrease the risk of heart disease.	[29]
Chaye sabz	Green tea	*Camellia sinensis*	Theaceae	Reduces inflammation, improves vascular function, and lowers cholesterol, thereby reducing the risk of cardiovascular disease.	[30]
Gole mohammadi	Damask Rose	*Rosa damascena*	Rosaceae	Reduces stress and inflammation in the blood vessels, improving circulation and reducing the risk for heart disease	[31]

The literature review indicates that medicinal plants such as marjoram, borage, lavender, valerian, hops, hawthorn, rosemary, chamomile, dracocephalum, mint, green tea, and damask rose are used for stress relief and disease management, with particular emphasis on cardiovascular disease (Table-2).

Table-1 catalogs several plants with diverse effects on the cardiovascular system and stress reduction. These plants come from various families, including Lamiaceae (mint, lavender, marjoram, dracocephalum), Rosaceae (hawthorn, damask rose), and other families such as Boraginaceae (borage), Valerianaceae (valerian), and Cannabaceae (hops).

They primarily exhibit effects such as sedation, anxiety reduction, anti-inflammatory properties, and blood pressure reduction. Notably, the Lamiaceae family makes up the largest portion of this table with four plants, and these plants have significant effects including stress reduction, blood pressure reduction, and anti-inflammatory properties. Other plants, such as green tea and borage, have anti-inflammatory and antioxidant effects that help reduce the risk of cardiovascular disease.

Rosehip, in addition to its sedative and anti-anxiety effects, helps reduce vascular inflammation and improve cardiovascular function. In summary, sedative, anxiolytic, and anti-inflammatory effects are common to most herbs, and many have demonstrated efficacy in lowering blood pressure and improving cardiovascular function. Herbs from the Lamiaceae family are particularly beneficial for stress reduction and blood pressure management, while green tea and rose hips offer pronounced benefits in reducing the risk of cardiovascular disease and improving cardiac function. This information is valuable in selecting effective herbs for the treatment of cardiovascular disease and stress reduction.

## Discussion

Medical approaches that incorporate traditional treatments have been shown to be particularly effective in managing stress and reducing the risk of CVD. One of the key aspects of these approaches is the use of medicinal plants to reduce stress, lower blood pressure, and improve heart health. This review examines the effects of medicinal plants in reducing stress and, consequently, the risk of cardiovascular disease.

Marjoram is known for its sedative and anxiolytic properties. Studies suggest that this plant, which acts as an anti-inflammatory and antispasmodic agent, may help reduce stress and high blood pressure. In patients with coronary artery disease, marjoram may help reduce chronic inflammation and improve overall cardiovascular health. In addition, its anti-anxiety effects help improve the quality of life for patients with hypertension and chronic stress [21]. Borage plays a key role in managing stress and reducing the risk of cardiovascular disease due to its anti-inflammatory and antioxidant properties [22].

This plant can help improve cardiac function and reduce vascular inflammation, thereby reducing the risk of cardiovascular disease. In addition, as a natural sedative, borage may help reduce anxiety and stress in patients [22]. Lavender, known for its calming and anxiolytic properties, has been shown to reduce stress and improve sleep quality. In patients with cardiovascular disease, reducing stress and anxiety may help lower blood pressure and improve coronary artery function. Research suggests that lavender may indirectly reduce the risk of cardiovascular disease by reducing inflammation and stress [23]. Valerian is known for its calming effects and effectiveness in reducing stress and improving sleep quality. Adequate sleep is a critical factor in maintaining cardiovascular health. For patients with cardiovascular disease, valerian may help lower blood pressure and stress, thereby preventing the progression of heart disease [24]. Hops are widely used for their sedative and anxiolytic effects. By affecting the central nervous system, this herb can help reduce stress and anxiety, thereby lowering blood pressure and vascular inflammation. In patients with cardiovascular disease, hops may help prevent vascular damage and improve heart function due to its anti-inflammatory properties [25].

Hawthorn is known for its ability to strengthen the heart and blood vessels. With its antioxidant and anti-inflammatory properties, this herb can improve vascular function and lower blood pressure. In patients with coronary artery disease, hawthorn may help reduce the risk of cardiovascular disease and improve quality of life [26].

Rosemary is known for its anti-inflammatory and cardiovascular properties. Consumption of rosemary may help reduce stress and improve cardiovascular function, thereby reducing the risk of cardiovascular disease. This herb benefits patients with cardiovascular disease by improving vascular health and strengthening the circulatory system [27]. Chamomile, known for its calming and anti-inflammatory properties, effectively reduces stress and blood pressure. In addition to its anti-inflammatory effects, chamomile serves as a natural sedative that can improve sleep quality and reduce anxiety in patients. Since inflammation and stress are significant contributors to the development of cardiovascular disease, the use of chamomile can be instrumental in managing these factors [28]. Dracocephalum, a plant with calming and anti-anxiety properties, helps reduce stress and blood pressure. It is particularly effective in reducing inflammation and improving cardiovascular health, reducing the risk of cardiac complications in patients with cardiovascular disease [28].

Mint, known for its calming and anti-inflammatory properties, can effectively reduce stress and improve blood circulation. Mint consumption is particularly beneficial in lowering blood pressure and improving heart function in patients with cardiovascular disease [29]. Green tea is recognized as a powerful antioxidant that reduces inflammation, improves vascular function, and lowers cholesterol levels. Regular consumption of green tea may prevent cardiovascular disease and reduce the risk of heart attack [30]. Damask rose, with its anti-inflammatory and calming effects, can reduce stress and vascular inflammation. This plant is particularly beneficial in improving cardiovascular function and reducing the risk of cardiovascular disease [31]. Chronic stress is a significant contributor to the development of cardiovascular diseases (CVD).

During stressful situations, the body releases stress hormones such as adrenaline and cortisol, which lead to an increase in heart rate and blood pressure. These physiological responses, when occurring repeatedly and chronically, can cause damage to the heart and blood vessels. Additionally, chronic stress can result in ongoing inflammation in the body, which over time can harm the walls of blood vessels, increasing the risk of atherosclerosis, heart failure, and other cardiovascular conditions [32][33]. In this context, the use of medicinal plants with stress-relieving and anti-inflammatory properties can help mitigate the negative effects of stress on the cardiovascular system. Herbs such as marjoram, chamomile, lavender, and borage assist in reducing stress and anxiety, thereby preventing elevated blood pressure. These plants also reduce inflammation, helping to protect the cardiovascular system from damage. Furthermore, plants like Damask rose and green tea, with their antioxidant effects, help reduce inflammation and improve heart function [34][35]. Thus, incorporating medicinal plants as natural supplements can play a significant role in alleviating stress, improving heart health, and lowering the risk of cardiovascular diseases.

## Conclusion

Herbal plants can be an effective strategy for reducing stress and improving cardiovascular health in patients with coronary artery disease. Due to their low risk and broad therapeutic benefits, these plants can enhance the quality of life for patients, especially when used alongside conventional treatments. This integrative approach can help reduce cardiovascular risk factors and improve the clinical condition of patients. Given the therapeutic potential of herbal plants, further research is needed to identify the precise mechanisms of their effects and to establish their clinical efficacy in managing cardiovascular diseases.

## Conflict of Interest

The authors have no competing interests to declare that are relevant to the content of this article.
